# De novo adult acute myeloid leukemia with two new mutations in juxtatransmembrane domain of the *FLT3* gene: a case report

**DOI:** 10.1186/s13256-020-02587-3

**Published:** 2021-01-26

**Authors:** Ismael F. Alarbeed, Abdulsamad Wafa, Faten Moassass, Bassel Al-Halabi, Walid Al-Achkar, Thomas Liehr, Imad Aboukhamis

**Affiliations:** 1 Department of Microbiology, Hematology and Immunology, Faculty of Pharmacy, Damascus University, Ministry of High Education, Damascus, Syria; 2 Department of Molecular Biology and Biotechnology, Human Genetics Division, Atomic Energy Commission, Damascus, Syria; 3 Jena University Hospital, Institute of Human Genetics, Friedrich Schiller University, Am Klinikum 1, 07747 Jena, Germany

**Keywords:** Acute myeloid leukemia, *FLT3*-ITDs, ITDs size, Sanger sequencing, Prognostic factors

## Abstract

**Background:**

Approximately 30% of adult acute myeloid leukemia (AML) acquire within fms-like tyrosine kinase 3 gene (*FLT3*) internal tandem duplications (*FLT3*/ITDs) in their juxtamembrane domain (JMD). *FLT3*/ITDs range in size from three to hundreds of nucleotides, and confer an adverse prognosis. Studies on a possible relationship between of *FLT3*/ITDs length and clinical outcomes in those AML patients were inconclusive, yet.

**Case presentation:**

Here we report a 54-year-old Arab male diagnosed with AML who had two *FLT3*-ITD mutations in addition to *NPM1* mutation. Cytogenetic approaches (banding cytogenetics) and fluorescence in situ hybridization (FISH) using specific probes to detect translocations t(8;21), t(15;17), t(16;16), t(12;21), and deletion del(13q)) were applied to exclude chromosomal abnormalities. Molecular genetic approaches (polymerase chain reaction (PCR) and the Sanger sequencing) identified a yet unreported combination of two new mutations in *FLT3*-ITDs. The first mutation induced a frameshift in JMD, and the second led to a homozygous substitution of c.1836T>A (p.F612L) also in JMD. Additionally a *NPM1* type A mutation was detected. The first chemotherapeutic treatment was successful, but 1 month after the initial diagnosis, the patient experienced a relapse and unfortunately died.

**Conclusions:**

To the best of our knowledge, a combination of two *FLT3*-ITD mutations in JMD together with an *NPM1* type A mutation were not previously reported in adult AML. Further studies are necessary to prove or rule out whether the size of these *FLT3*-ITDs mutations and potential other double mutations in *FLT3*-ITD are correlated with the observed adverse outcome.

## Background

In patients with acute myeloid leukemia (AML) genetic diagnostics were performed in the past mainly by cytogenetics and molecular cytogenetics. In recent years also tumor markers were added, which rely on molecular genetic methods [1].

The fms-like tyrosine kinase 3 (*FLT3*) gene encodes a class III tyrosine kinase receptor for the FLT3 ligand, which is normally expressed in CD34^+^‏ hematopoietic stem/progenitor cells, and plays a fundamental role in both normal and leukemic hematopoiesis [2]. Internal tandem duplications (ITDs) of the *FLT3* gene (*FLT3*/ITDs) represent one of the most common molecular abnormalities in patients with AML. They are detectable in around 25–30% of all patients [3, 4]. ITDs consist of in-frame insertions of duplicated sequences localized in the juxtamembrane domain (JMD) of the FLT3 molecule. Their presence results in a constitutive, ligand independent activation of the tyrosine kinase activity of the FLT3 receptor; this is responsible for abnormal proliferation and differentiation of leukemic stem cells [2]. In AML constitutive activation of kinase domain happens due to disruption of auto-inhibitory interaction between JMD and the activation loop, which normally stabilizes the inactive kinase, and at the same time protects ATP binding pocket [5, 6]. *FLT3*/ITDs also protect leukemic cells from the damaging chemotherapeutic agents [7].

It was suggested that an increasing length of additional sequences like ITDs may influence the grade of tyrosine kinase activity of the FLT3 receptor, and could (1) lead progressively to increasing activation levels and (2) to worsen the overall survival (OS) in affected patients. Still, the results of studies which investigated the impact of ITD length on the clinical outcome are contradictory. Some studies confirming the aforementioned assumption [8–12], whereas others contradicted [13, 14].

Presence of *FLT3*/ITDs has been associated with an increased initial peripheral white blood cell (WBC) count and percentage of blast cells in bone marrow, a reduced disease-free survival (DFS) and OS, and a high relapse rate with an overall adverse prognosis. However, the rate of complete remission (CR) was not significantly affected [15–17]. Thus, a prognostic significance of *FLT3*/ITDs has been suggested [8]. According to the National Comprehensive Cancer Network and the European LeukemiaNet (ELN) 2017, AML cases with cytogenetically normal karyotypes and *FLT3*/ITD mutation have a poor prognosis.

Besides in *FLT3*/ITD, mutations in nucleophosmin 1 (*NPM1*) gene represent the second most frequent molecular aberration in AML patients [18]. A combined status of mutated *NPM1* and the wild type *FLT3* gene (*NPM1*^+^/*FLT3*^−^) is a well-established favorable risk factor in younger adult patients, with less probability of relapse and prolonged survival [19–21]; these patients are not obliged to receive allogeneic hematopoietic stem cell transplantation [22]. Otherwise, co-occurrence of *FLT3*/ITD and *NPM1* mutations was suggested to partially improve response rates, DFS and OS outcomes compared to AML-patients having exclusively *FLT3*/ITD mutations. However, cases with mutations in *FLT3*/ITD and *NPM1* have worse prognosis than those having (*NPM1*^+^/*FLT3*^−^) [23].

Here, we present a unique case with two *FLT3*-ITDs mutations in JMD and an *NPM1* type A gene mutation associated with adverse outcome.

## Case presentation

In October 2019, a 54-year-old Arab male patient presented with 2 months history of fatigue, orthostatic hypotension followed by bruising on the lower right extremity, melena (present for one month only) and dyspnea II. Physical examination and computer tomographic scan showed hepatomegaly (4 cm). He had no familial history of malignancies and no social and environmental history of exposure to toxins or animals. Initial laboratory evaluation of peripheral blood (PB) revealed white blood cells count (WBC) of 26.3 × 10^9^/l (10% were blasts). Pathologic examination of bone marrow (BM) aspirate characterized hybercellularity with 60% of blasts. Flow cytometric (FCM) analysis classified this case as AML-M2 according to world health organization (WHO) classification. The abnormal cell population (60%) was positive for CD45^dim^, CD34, HLADr, CD13, CD33 and expressed CD117 heterogeneously. Blasts cell population was negative for CD3, CD117, CD14, cCD3, cCD79a, CD14, CD11c, CD38, CD64, CD32, CD7, CD19, CD10, and CD5.

The patient was given standard treatment for AML including 3 + 7 induction chemotherapy (daunorubicin 60 mg/m^2^ for 3 days and cytarabine 200 mg/m^2^ for 7 days). One month later, under treatment with 3 + 7 protocol, the patient relapsed, i.e. his PB showed a WBC of 107 × 10^9^/l, anemia (hemoglobin level (Hgb) = 8.8 g/dl) and thrombocytopenia (Plt 93 × 10^9^/l). The patient was given re-induction with 3 + 7 chemotherapy protocol (for more details see Table 1). Less than one month after relapse, the patient acquired additional severe symptoms such as neutropenia, neutropenic enterocolitis, and diabetes insipidus, and the patient unfortunately passed away due to respiratory and cardiac arrest. No autopsy was performed. The patient’s brother agreed with the scientific evaluation of this case and the study was approved by the ethical committee of Pharmacy faculty at Damascus University, Ministry of High Education, Syria review Board, No. 2/2019.

**Table 1:** Clinical history of the patient together with diagnostic results and treatment

Day of treatment	Symptoms	Analysis findings	Treatment and outcomes
1		Serum biochemistry analyses: Calcium (Ca^2+^) 8.3 mg/dL (normal value 8.5–10.6) Lactate dehydrogenase (LDH) 558 U/L (normal level < 460) Phosphor 4.4 mg/dL (2.7–6) Uric acid (UA) 5.6 mg/dL (normal value 3.5–7) Creatinine (creat.) 0.7 mg/L (normal 0–5) Urea 17 mmol/L (normal 10–50) Sodium (Na^+^) 135 mmol/L (normal135–148) Potasium (K^+^) 4.5 mmol/L (3.5–5.2) Total protein (TP) 8 g/dL (normal 6.6–8.7) Albumin (Alb) 4.4 g/dL (normal 3.8–5.4) Bilirubine 5.1 mg/dL (normal 0–5) Glucose 101 mg/dL (normal 65–110). Prothrombin time (PT) 85% Partial thromboplastin time (PTT) 28.5 seconds International normalized ratio (INR) 1.1 seconds C-reactive protein (CRP) 43.8 mg/L (normal 0–5)	D1 of (3+7) protocol: Daunorubicin 60 mg/m^2^ for 3 days and Cytarabine 200 mg/m^2^ for 7 days Blood transfusion
2		Glucose 166 mg/dL (normal 65–110)	Patient was developed fever (39 °C), epigastric burning pain, no diarrhoea, cough with white mucus and pharyngeal congestion Blood transfusion
7	PB showed WBC 3.9 × 10^9^/L, anemia (Hgb 7.8 g/dL); thrombocytopenia (Plt 24 × 10^9^/L)	Creat. 1.7 mg/L (normal 0–5) Urea 23 mmol/L (normal 10–50) Glucose 141 mg/dL (normal 65–110)	Blood transfusion
13	Patient was developed neutropenia (WBC 0.6 × 10^9^/L), anemia (Hgb 7 g/dl); thrombocytopenia (Plt 2 × 10^9^/L)	Creat. 0.9 mg/L Urea 40.9 mmol/L Glucose 117.4 mg/dL UA 2.7 Ca^2+^ 8.3 Phosphor 2.1 mg/dL (2.7-6) Alanine aminotransferase (ALT) 25.3 U/L (normal 0–45) Aspartate aminotransferase (AST) 51.5 U/L (normal 0–35)	Blood transfusion and broad-spectrum antibiotics
30	Patient was relapsed His PB showed: WBC 107 × 10^9^/L, anemia (Hgb 8.8 g/dL); thrombocytopenia (Plt 93 × 10^9^/L) BM smear showed almost 40% of blasts Submandibular lymphadenopathy (2 cm), fever (39.5 °C), cough with white mucus, and severe diarrhea Heart rate 89/minute	Ca^2+^ 9.2 mg/dL LDH 833 U/L UA 6.2 mg/dL Creat. 1 mg/L Urea 10 mmol/L Na^+^ 131 mmol/L K^+^ 3.2 mmol/L TP 6.2 g/dL PT 46% INR 1.6 seconds ALT 10.6 U/L AST 19.5 U/L	Re-induction (3 + 7) protocol Patient was developed sever neutropenia (WBC 0.5 × 10^9^/L), anemia (Hgb 7.1 g/dL); thrombocytopenia (Plt 9 × 10^9^/L) Blood transfusion and broad-spectrum antibiotics
38	Patient was developed sever neutropenia (WBC 0.0 × 10^9^/L) was continues for 2 days later, anemia (Hgb 6.5 g/dL); thrombocytopenia (Plt 1 × 10^9^/L) Fever (39–39.5 °C) was continues for the next 7 days	K^+^ 3.3 mmol/L CRP 300 mg/L Procalcitonine 68 ng/mL (normal 0.1–0.49)	Neutropenia, abdominal pain, right iliac fossa pain, and severe diarrhoea Blood transfusion and broad-spectrum antibiotics Abdomen CT scan showed: Multi-focal of splenic lesions (2 cm) which consistent with secondary metastasis, ascending colon wall thickness (1.7 cm), cecum wall thickening until the appendix (1.5 cm) with fatty infiltration which is consistent with neutropenic enterocolitis, Paraaortic lymph node enlargement (0.8 cm), bone scan shows degenerative changes and free fluid in abdomen and fatty infiltration (appendicitis).
40	His PB showed: WBC 1.5 × 10^9^/L, anemia (Hgb 6.1 g/dL); thrombocytopenia (Plt 1 × 10^9^/L)	PT 71% INR 5.3 seconds K^+^ 2.6 mmol Glucose 128 mg/dL Urea 56 mmol/L Creat. 0.6 mg/L Na^+^ 152 mmol/L	Blood transfusion and broad-spectrum antibiotics
46	He suffered from fever more than 39–39.5 °C and neutropenia for more than 7 days		Approximately 3 months after initial diagnosis he died due to respiratory and cardiac arrest No autopsy was performed

Ca^2+^, Calcium; LDH, Lactate dehydrogenase; UA, Uric acid; creat., Creatinine; Na^+^, Sodium; K^+^, potassium; TP, Total protein; Alb, Albumin ; PT, Prothrombin time; PTT, Partial thromboplastin time; INR, International normalized ratio; CRP, C-reactive protein; ALT, Alanine aminotransferase; AST, Aspartate aminotransferase; WBC, white blood cells; PB, peripheral blood; Hgb, hemoglobin; BM, Bone marrow; CT, computed tomography scan.

Chromosome analysis using GTG-banding was performed on BM sample taken prior to chemotherapy according to standard protocols [24]. A normal male karyotype was diagnosed. Fluorescence in situ hybridization (FISH) using specific probes to detect translocations t(8;21), t(15;17), t(16;16), t(12;21), and deletion del(13q), were applied to excluded chromosomal abnormalities, too, as previously reported [24].

**Fig. 1:**
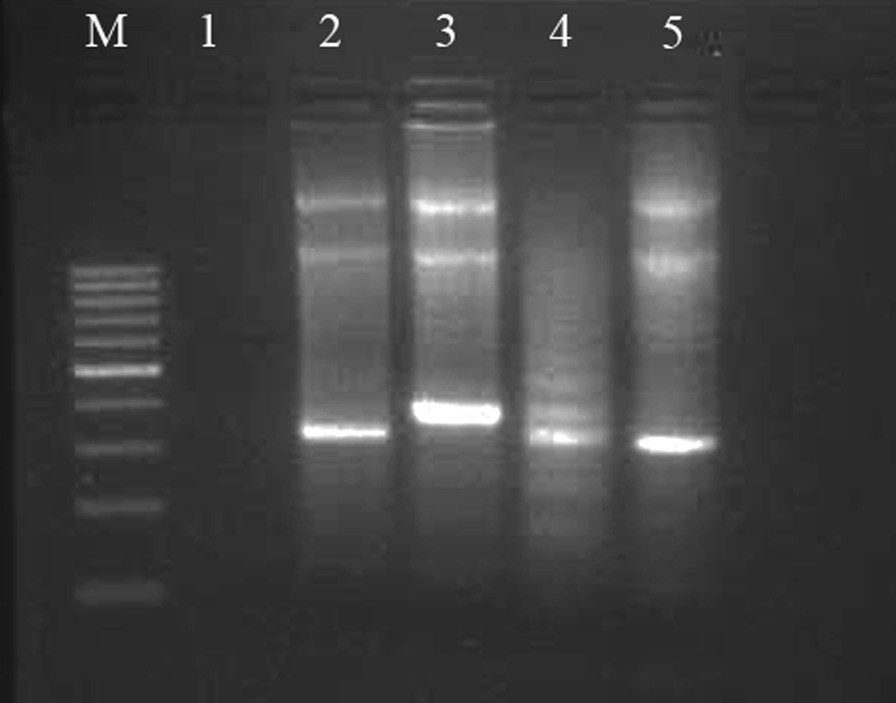
Agarose gel electrophoresis. The PCR amplification products of ITD. M indicates the molecular weight marker (100 bp); line 1, blank PCR products; lines 2 and 5, wild-type *FLT3*-ITD; line 3, the band of 390 bp in our patient; and line 4, mutant *FLT3*-ITD.

**Fig. 2:**
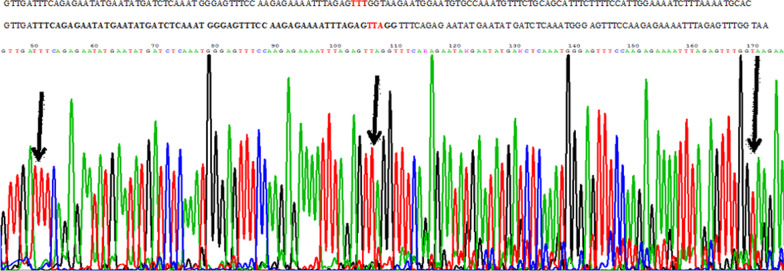
Sanger sequence of the ITD mutation, revealed an insertion and a duplicated mutation sequence, respectively.

For molecular analyses, whole genomic DNA was extracted from PB cells (EDTA-blood) prior to chemotherapy treatment. Polymerase chain reaction (PCR) amplification of genomic DNA and Sanger sequencing were used to screen for the presence of mutations of the following genes: *FLT3*/ITD (exons 11 and 12), *FLT3*-KTD and *NPM1*; using specific primers for each mutation previously reported [25]. ITDs were confirmed by Sanger sequence analysis; the wild-type band of 330 bp length, and other differently sized PCR products were identified in our patient (Fig. 1) using the ABI Prism 310 genetic analyzer (Applied Biosystems, Foster City, CA, USA). Two novel frameshift mutations of the JMD in *FLT3*-ITD were identified in our patient (see also Fig. 2):

mutation 1: c.1779-1780insTTTCAGAGAATATGAATATGATCTCAAATGGGAGTTTCCAAGAGAAAATTTAGAGTTAGG (p.D593-F594insREYEYDLKWEFPRENLEF).

mutation 2: homozygous substitution c.1836T>A (p.F612L).

A D835 mutation was not detected by *FLT3*-KTD test in our patient. However, he had also *NPM1* type A mutation (data not shown).

## Discussion and conclusions

Here we report the first case of an adult AML patient with normal karyotype, who had one *NPM1* type A and two frameshift *FLT3*-ITD mutations. The first frameshift *FLT3*-ITD was never reported before (COSMIC database for somatic samples from hematopoietic and lymphoid tissue), whereas the second mutation has already been observed, but as heterozygous variant (COSV54057677).

The present case supports previous findings [8–12], which suggested that long ITDs are associated with adverse OS, a higher incidence for relapse and a negative impact on clinical outcomes in AML patients post chemotherapy.

Of special interest, is the suggestion that the complications of intense chemotherapy, as observed in our patient (see Table 1), could also have been promoted by the observed combination of mutation events. The observed neutropenia, is associated with the risk for developing serious and complicated infections or even sepsis [26–28]. Also neutropenic enterocolitis (NE), a necrotizing process usually localized to the ascending colon, cecum, and terminal ileum [29, 30] can appear in 15% of AML patients treated with a combination of Idarubicin and Cytosine arabinoside [31], It also associated with increased mortality [32]. Finally, central diabetes insipidus (DI) is a rare complication in AML and myelodysplastic syndrome (MDS) cases with less than 100 cases reported in literature [33]. Central DI can precede the diagnosis of AML or MDS or it can manifest during treatment and was thought to confer a poor prognosis [33]. The pathogenesis of central DI in AML and MDS may be secondary to leukemic infiltration of the infundibulum, hemorrhage, thrombosis, infection, or autoimmunity [34].

Further studies are needed to prove or rule out whether the size of the *FLT3*-ITDs mutation and the double mutations in *FLT3*-ITD are correlated with an adverse prognosis. Also, more research is needed to see if chemotherapy-complications as observed here can be omitted by the application of other treatment regimes.

## Data Availability

All relevant data and material is included in this publication.

## Abbreviations

AML: Acute myeloid leukemia
BM: Bone marrow
CR: Complete remission
DFS: Disease-free survival
DI: Diabetes insipidus
FISH: Fluorescence in situ hybridization
FCM: Flow cytometric
FLT3: Fms-like tyrosine kinase 3 gene
JMD: Juxtamembrane domain
ITDs: Internal tandem duplications
MDS: Myelodysplastic syndrome
NPM1: Nucleophosmin 1 gene
NE: Neutropenic enterocolitis
OS: Overall survival
PB: Peripheral blood
WBC: White blood cells
WHO: World health organization
